# Win, Lose, or Tie: Mathematical Modeling of Ligand Competition at the Cell–Extracellular Matrix Interface

**DOI:** 10.3389/fbioe.2021.657244

**Published:** 2021-04-29

**Authors:** Zeynep Karagöz, Thomas Geuens, Vanessa L. S. LaPointe, Martijn van Griensven, Aurélie Carlier

**Affiliations:** Department of Cell Biology–Inspired Tissue Engineering, MERLN Institute for Technology-Inspired Regenerative Medicine, Maastricht University, Maastricht, Netherlands

**Keywords:** integrin, ligand competition, computational model, extracellular matrix, ordinary differential equation

## Abstract

Integrin transmembrane proteins conduct mechanotransduction at the cell–extracellular matrix (ECM) interface. This process is central to cellular homeostasis and therefore is particularly important when designing instructive biomaterials and organoid culture systems. Previous studies suggest that fine-tuning the ECM composition and mechanical properties can improve organoid development. Toward the bigger goal of fully functional organoid development, we hypothesize that resolving the dynamics of ECM–integrin interactions will be highly instructive. To this end, we developed a mathematical model that enabled us to simulate three main interactions, namely integrin activation, ligand binding, and integrin clustering. Different from previously published computational models, we account for the binding of more than one type of ligand to the integrin. This competition between ligands defines the fate of the system. We have demonstrated that an increase in the initial concentration of ligands does not ensure an increase in the steady state concentration of ligand-bound integrins. The ligand with higher binding rate occupies more integrins at the steady state than does the competing ligand. With cell type specific, quantitative input on integrin-ligand binding rates, this model can be used to develop instructive cell culture systems.

## Introduction

The extracellular matrix (ECM) is a mesh of fibrous proteins that forms the basis of the tissue architecture and structurally supports the cells. The translation of biophysical cues provided by the ECM into biochemical signals by the cells is a process called mechanotransduction. For cells, mechanotransduction is central to maintaining homeostasis in many biological processes like proliferation, migration, differentiation, and apoptosis (Miller et al., 2020). It is known, for example, that the composition and mechanical properties of the extracellular environment in which mesenchymal stem cells are grown influences whether they differentiate into adipocytes, osteoblasts, or chondrocytes (Assis-Ribas et al., 2018). When mechanotransduction is disturbed, it results in aberrant cell behavior and thus impaired tissue function (Handorf et al., 2015).

Focal adhesions are multiprotein complexes where this mechanotransduction process is orchestrated. The main players in focal adhesions, responsible for physical interactions with the ECM, are integrins. Each integrin consists of non-covalently associated α and β subunits. To date, 24 unique integrins have been found in humans, which are combinations of 18 different α and eight different β subunits (Hynes, 2002; Barczyk et al., 2010). Each integrin heterodimer is able to recognize and bind to a defined set of ECM ligands via its ectodomain (Hynes, 2002; Humphries et al., 2006). Different ligand-bound integrins can further form clusters amongst each other via non-covalent links between α and β subunits. Approximately 50 integrins can cluster together (Changede et al., 2015). This way, integrins create physical anchor points between the extracellular space and the cytoskeleton and initiate the focal adhesion formation. Cytosolic ligands are recruited to cytoplasmic tails of integrin molecules, and mechanosensitive signaling is activated in the cell via the focal adhesions (Hynes, 2002).

Due to the broad range of cellular response activated via integrin-mediated signaling, integrins have been targets for tissue engineering applications. Recent developments in methods that make use of stem cells and targeted differentiation protocols, such as in organoid development, demonstrated the importance of a detailed understanding of mechanotransduction and particularly integrin–ECM interactions. So called “designer matrices” that are decorated with integrin-binding partners and that are adaptive in terms of their mechanical properties have been shown to enhance intestinal organoid culture survival and proliferation (Gjorevski et al., 2016). Similarly, by mimicking the physiological environment of early stages of embryonic development in cell culture, the formation of human pluripotent stem cell–derived kidney organoids could be enhanced (Garreta et al., 2019).

Maintaining the appropriate ECM composition is critical for kidney organoid development. For example, Geuens et al. (2020a) reported an unwanted increase in specific ECM proteins when cell culture times were prolonged in an attempt to increase kidney organoid maturation. They performed a tandem mass spectrometry analysis to compare the ECM composition of kidney organoids that were cultured for 18 and 25 days. Older kidney organoid ECM was rich in collagens (specifically COL1A1, COL2A1, and COL6A1) and fibronectin, which are hallmarks of tissue fibrosis, compared to day 18 ECM. The analysis also showed an increase in αSMA — a myofibroblast marker — in older kidney organoids, that further indicated tissue fibrosis. Following this analysis, they encapsulated the kidney organoids in a soft hydrogel system, which prevented the unwanted ECM deposition, perhaps by better mimicking the natural environment in kidney development (Geuens et al., 2020a).

The effect of the abnormal accumulation of particular ECM proteins on cell phenotype is worth exploring for the future of organoid culture systems. The initial presence and the changes in the amounts of ECM proteins are sensed first by the integrins, the direct interaction partners of these proteins. Therefore, a detailed analysis and understanding of the effects of abnormal ligand deposition and ligand competition on integrin–ligand dynamics can help us understand the decision-making processes of the cells in response to the changes in ECM conditions (Garreta et al., 2019; Miller et al., 2020; Geuens et al., 2020b).

**FIGURE 1:**
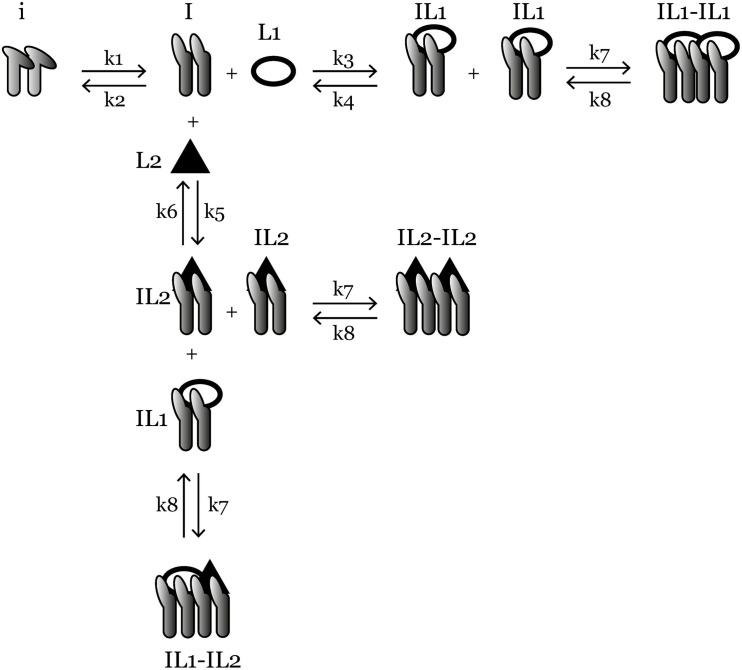
An overview of the ligand-competition model reactions. *i* is the inactive integrin, *I* is activated integrin, *IL1* is *L*1-bound integrin, *IL*2 is *L*2-bound integrin, *IL1-IL1*, *IL2-IL2*, and *IL1-IL2* are three species of clustered integrins with different ligand compositions. *k*1–*k*8 are reaction rate constants and their values are given in Table 1. Ordinary differential equations representing the reactions are given in the “Materials and Methods” section.

The high number of potential integrin–ligand pairs make it difficult to test and document the effects experimentally. Therefore, computational modeling provides a unique opportunity for exploring the integrin–ligand binding process and its subsequent effects. There exists a number of computational models that explain different processes in the integrin-related pathways. In particular, Hudson et al. (2017) studied the binding of fibronectin and von Willebrand Factor A (vWA) to integrin α_v_β_3_ as well as binding of collagen to α_1_β_1_ using an ordinary differential equation (ODE) model; they reported an increase in ligand-bound integrin at the steady state when there is an increase in the concentration of ligands. However, they simulate the integrin–ligand binding exclusively for each ligand, which overlooks the fact that the ligands of the same integrin are in a competition to bind when present at the same time. To fill this knowledge gap and identify potential patterns in integrin–ligand binding that occur due to the competition between multiple ligands for the same integrin, we developed an ODE model. Our model consists of three reaction levels: (1) integrin activation, (2) ligand binding, and (3) ligand-bound integrin clustering (Figure 1). Using this model, we explore the changes in ligand-binding kinetics when the amount of ECM ligands changes over time, as in the case of kidney organoid cultures.

## Materials and Methods

### Ordinary Differential Equation Model

We used the Tellurium Python environment (Choi et al., 2018) to generate and the libRoadRunner library (Somogyi et al., 2015) to simulate the ligand competition model. The python code for the model and simulations as well as the SBML file for the model and the simulation results in csv format can be found in our GitHub repository^1^.

The mass-action kinetics scheme of the integrin–ligand competition model is given in Figure 1; here we present the differential equations for the model (Eqs 1–9): where *i* denotes the concentration of inactive integrins, *I* is the concentration of active integrins, *IL1* and *IL2* are the concentrations of *L1*- and *L2*-bound integrins, respectively. *C1*, *C2*, and *C3* are the concentrations of three distinct types of integrin clusters composed of *IL1-IL1*, *IL2-IL2*, and *IL1-IL2*, respectively. *k*1−*k*8 are the reaction rate constants of the reversible reactions in the model (Figure 1) and their values are given in Table 1.

**TABLE 1:** Parameters used in the ODE model, their values and references.

**Model parameter**	**Explanation**	**Value**	**References**
*k*1	Integrin α_v_β_3_ activation	5 × 10^6^ 1/(nM × s)	Yu et al., 2017
*k*2	Integrin α_v_β_3_ inactivation	1.0 × 10^8^ 1/s	Yu et al., 2017
*k*3	Fibronectin (*L*1) – α_v_β_3_ binding	1.6 × 10^8^ 1/(nM × s)	Hudson et al., 2017
*k*4	Fibronectin (*L*1) – α_v_β_3_ unbinding	3.5 × 10^–1^ 1/s	Hudson et al., 2017
*k*5	vWA (*L*2) – α_v_β_3_ binding	1.6 × 10^4^ 1/(nM × s)	Hudson et al., 2017
*k*6	vWA (*L*2) – α_v_β_3_ unbinding	2.3 × 10^–2^ 1/s	Hudson et al., 2017
*k*7	Integrin cluster formation	1.6 × 10^8^ 1/(nM × s)	Yu et al., 2017
*k*8	Integrin cluster dissociation	0.5 × 10^7^ 1/s	Yu et al., 2017
*i*	Integrin α_v_β_3_	0.05 nM	Hudson et al., 2017


(1) $$\frac{d\,i}{d\,t}=-k\,1\times i+k\,2\,\times I$$

(2) $$\frac{d\,I}{d\,t}=k\,1\,\times i-k\,2\,\times I-k\,3\times I\times L\,1+k\,4\times I\,L\,1-k\,5\times I\times L\,2+k\,6\times I\,L\,2$$

(3) $$\frac{d\,I\,L\,1}{d\,t}=k\,3\times I\times L\,1-k\,4\times I\,L\,1-2\times \left(k\,7\times I\,L\,1^{2}-k\,8\,\times C\,1\right)-k\,7\times I\,L\,1\,\times I\,L\,2+k\,8\times C\,3$$

(4) $$\frac{d\,I\,L\,2}{d\,t}=k\,5\times I\times L\,2-k\,6\times I\,L\,2-2\times \left(k\,7\times I\,L\,2^{2}-k\,8\times C\,2\right)-k\,7\times I\,L\,1\times I\,L\,2+k\,8\times C\,3$$

(5) $$\frac{d\,C\,1}{d\,t}=k\,7\times I\,L\,1^{2}-k\,8\times C\,1$$

(6) $$\frac{d\,C\,2}{d\,t}=k\,7\,\times I\,L\,2^{2}-k\,8\times C\,2$$

(7) $$\frac{d\,C\,3}{d\,t}=k\,7\times I\,L\,1\times I\,L\,2-k\,8\,\times C\,3$$

(8) $$\frac{d\,L\,1}{d\,t}=-k\,3\,\times I\times L\,1+k\,4\times I\,L\,1$$

(9) $$\frac{d\,L\,2}{d\,t}=-k\,5\,\times I\times L\,2+k\,6\times I\,L\,2$$

The inspiration for this model was a prior integrin–ligand binding model presented by Hudson et al. (2017). However, their model included only one type of ligand available at a time for one integrin type. We have modified this model to account for the competition of multiple ligands binding to the same integrin. We have also added an integrin activation step, before the initiation of ligand binding. This was to accommodate the conformational change (from bent to extended) of the integrin ectodomain, required for the ligand binding site to become available (Takagi and Springer, 2002; Zhu et al., 2008; Li and Springer, 2017). The rate of the activation step was calculated by Yu et al. (2017) using the energy required for the bent-to-extended conformation change (Huang et al., 2012; Yu et al., 2017). It should be noted that we do not make the distinction between the next two possible conformations (extended-closed and extended-open) after the ligand is bound to the integrin (Zhu et al., 2008; Li and Springer, 2017), as the switch between these two states is highly related to the integrin cytoplasmic tails binding to cytoskeleton, which is out of the scope of this study. Since there are two types of ligand-bound integrins in our model (*IL*1 and *IL*2), we also make the distinction of three possible integrin clusters (*C*1, *C*2, and *C*3). However, we assumed the cluster association/dissociation rate constants (*k*7 and *k*8, respectively) for distinct cluster types are the same, simply because the molecules that are interacting, the integrins, are of the same type for each cluster.

We used the binding rate constants of fibronectin and vWA to integrin α_v_β_3_ as the *L*1 and *L*2 binding rate constants (Table 1). We chose to model integrin α_v_β_3_ because of its relevance in kidney fibrosis (Henderson et al., 2013; Conroy et al., 2016; Bülow and Boor, 2019). Similarly, we chose fibronectin as the first ligand (*L*1) as its expression is related to fibrosis (Eddy, 1996; Genovese et al., 2014) and it is a relatively well characterized ligand of integrin α_v_β_3_ (Humphries et al., 2006). We used vWA as the second α_v_β_3_-binding ligand (*L*2) of which we derived the binding rate constant from a previous model (Hudson et al., 2017). Overall, we intended to demonstrate the simplest possible case of ligand competition where a low affinity and a high affinity ligand compete for binding to the integrin. *L*1 represents a high affinity ligand, whereas *L*2 represents a medium to low affinity ligand for integrin α_v_β_3_ (Irvine et al., 2002). This setup is relevant for natural cell-ECM interactions as well as for cells on synthetic substrates as one of the most widely used integrin-targeting peptide sequences RGD has varying affinities in different conformations (i.e., higher affinity when cyclic form vs lower affinity when in linear form; Xiao and Truskey, 1996; Verrier et al., 2002; Sankaran et al., 2017). When provided with the necessary parameter set, our model can be used to simulate the interactions of other integrin–ligand pairs or even other receptor–ligand pairs which have similar activation-binding-clustering chemistry.

### Design of *in silico* Experiments

**FIGURE 2:**
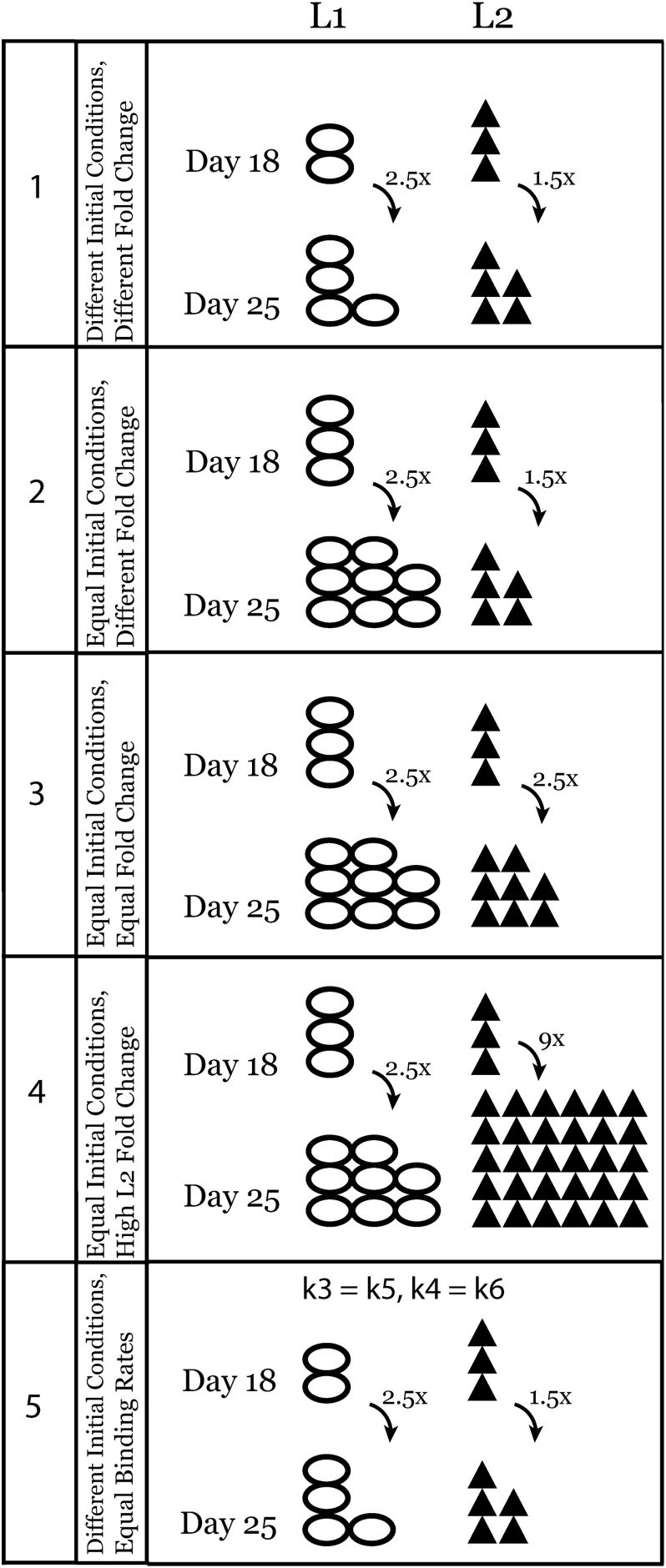
Schematic representation of experimental conditions that were tested using the ODE model of ligand competition. Conditions are numbered from 1 to 5 and this numbering is also used in the figures and results.

Using the ODE system described above, we performed a set of *in silico* experiments, aimed at characterizing the effects of increased ECM ligand concentration on integrin binding in a cell culture system. Initial molar concentrations for fibronectin (0.18 nM) and vWA (0.33 nM) were taken from Hudson et al. while we used the fold changes reported by Geuens et al. (2020a) between days 18 and 25 for these two ligands (2.5-fold and 1.5-fold for fibronectin and vWA) in kidney organoid culture. This way we obtained the test condition 1 “Different Initial Conditions, Different Fold Change” in Table 2 and Figure 2.

**TABLE 2:** Conditions (1–5) with initial concentration values for competing ligands, for each experiment [different or equal initial conditions (IC); different, equal or high fold change (FC) between experiment days; different or equal binding rates (BR) for ligands] and for each time point (Day 18 and 25).

	**Test condition**	**Experiment time**	***L*1**	***L*2**
1	Different initial conditions, Different fold change	Day 18	0.18 nM	0.33 nM
		Day 25	0.46 nM	0.50 nM
2	Equal initial conditions, Different fold change	Day 18	0.33 nM	0.33 nM
		Day 25	0.84 nM	0.50 nM
3	Equal initial conditions, Equal fold change	Day 18	0.33 nM	0.33 nM
		Day 25	0.84 nM	0.84 nM
4	Equal initial conditions, High fold change for *L*2	Day 18	0.33 nM	0.33 nM
		Day 25	0.84 nM	2.97 nM
5	Different initial conditions, Equal binding rates (*k*3 = *k*5 = 1.6 × 10^8^ 1/nM × s, *k*4 = *k*6 = 3.5 × 10^–1^ 1/s)	Day 18	0.18 nM	0.33 nM
		Day 25	0.46 nM	0.50 nM

In further *in silico* experiments, we set the initial concentrations of the two ligands equal (condition 2, Table 2 and Figure 2) and varied the fold change values (Equal, Different, or High Fold Change for *L*2 only) between days 18 and 25 to test their effect on the system (conditions 3 and 4, Table 2 and Figure 2). Finally, we set the binding rates of the two ligands to be equal and used the initial concentration of test condition 1 once more to see the effect of binding rate constants independent of the effect of initial ligand concentrations and fold changes (condition 5, Table 2 and Figure 2). A schematic representation of all the tests is given in Figure 2.

## Results

Using our ODE-based model (Eqs 1–9) and interactions described above (Figure 1), we performed simulations using reaction rate constants from the literature (Table 1) and initial conditions changing according to Table 2 and Figure 2. The binding rate constant between integrin α_v_β_3_ and fibronectin is 10^4^ times higher than that of integrin α_v_β_3_ and vWA (Hudson et al., 2017). Therefore, in our model, L1 is the ligand with higher binding affinity while *L*2 has lower binding affinity for the same integrin. Although we use binding rate constants of fibronectin and vWA in this study, the computational model is generic and can be adapted for other integrin–ligand pairs by changing the corresponding parameter values.

The initial conditions for the integrin and both ligands at day 18 were set from Hudson et al. (2017). The day 25 initial conditions for the two ligands were determined using the results of the kidney organoid ECM proteomics analysis (Table 2). It should be noted that in all *in silico* tests that are described in the following sections, the inactive integrin concentration (*i*) and active integrin concentration (*I*) reached a steady state value of almost zero. This means that all integrins in the system were found as either bound to a ligand and/or clustered with other ligand-bound integrins (Supplementary Figure 1).

### The Ligand With a Higher Binding Rate Dominates the Integrin Binding Competition

**FIGURE 3:**
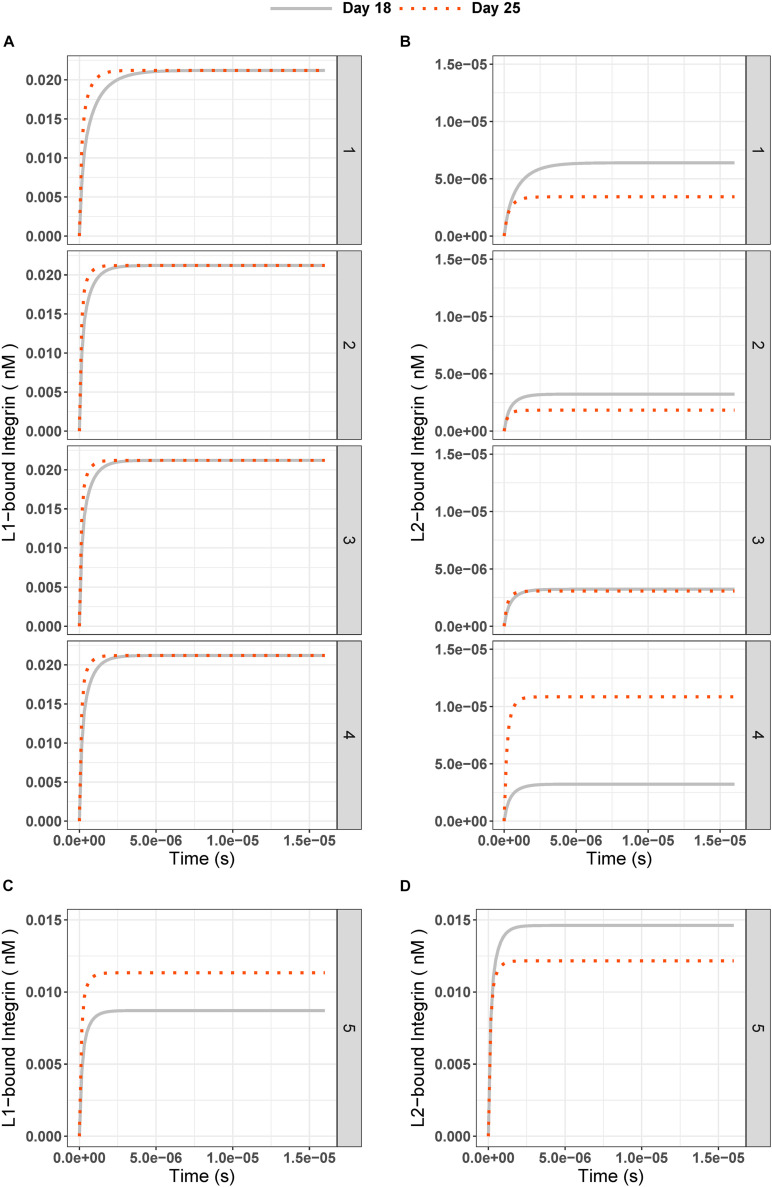
**(A)***L*1-bound and **(B)***L*2-bound integrin concentrations over time in experiment days 18 (gray solid line) and 25 (red dotted line) for test conditions 1 to 5. The test conditions are as given in Table 2 and Figure 2: (1) Different IC are 0.18 nM for *L*1, 0.33 nM for *L*2; (2) Different FC are 2.5 for *L*1, 1.5 for *L*2; (3) Equal IC is 0.33 nM; Equal FC is 2.5; and (4) High FC for *L*2 is 9. **(C)** *L*1-bound and **(D)** *L*2-bound integrin concentration over time for test condition 5 (Different IC and equal binding rate constants for ligands; *k*3 = *k*5 = 1.6 × 10^8^ 1/(nM × s), *k*4 = *k*6 = 3.5 × 10^–1^ 1/s).

First, we looked at how integrin–ligand binding dynamics change under conditions similar to the kidney organoid culture experiments. The proteomics analysis revealed that there was a 2.5– and 1.5–fold increase in the amount of fibronectin and vWA, respectively, between days 18 and 25 in the ECM. In our model, we simulated this scenario as condition 1, using the initial concentrations given in Table 2. The steady-state value was higher at day 25 than day 18 for *L*1-bound integrins (increase in the 10^–6^th order, Figure 3A-1, and Supplementary Figure 2A). The *L*2-bound integrin concentration, however, decreased at day 25 compared to day 18 (Figure 3B-1).

Second, to test the effect of the differences in initial conditions of the ligands (*L*1 = 0.18 nM and *L*2 = 0.33 nM) on our observations, we ran simulations with equal initial concentrations (0.33 nM) for *L*1 and *L*2 on day 18 and applied the same fold changes (Figures 3A-2,B-2). Similar results were found for *L*1-bound integrin, whereas *L*2-bound integrin had an even lower steady-state concentration (Day 18: 2.98 × 10^–6^ nM, Day 25: 1.77 × 10^–6^ nM, and Figure 3B-2) compared to those from Different IC simulations (Day 18: 5.46 × 10^–6^ nM, Day 25: 3.24 × 10^–6^ nM, and Figure 3B-1).

Third, we tested whether an equal fold change (2.5 for both *L*1 and *L*2, Figures 3A-2,B-3) between days 18 and 25 affected the ligand competition. The steady state for *L*1-bound integrin did not change compared to previous test (Figure 3A-3 and Supplementary Figures 2B,C). However, we observed an increase in the steady state of *L*2-bound integrins on day 25 (2.98 × 10^–6^ nM, Figure 3B-3) compared to that under Different FC conditions (1.77 × 10^–6^ nM, Figure 3B-2). This hinted that the fold change can affect the ligand competition in favor of *L*2.

**FIGURE 4:**
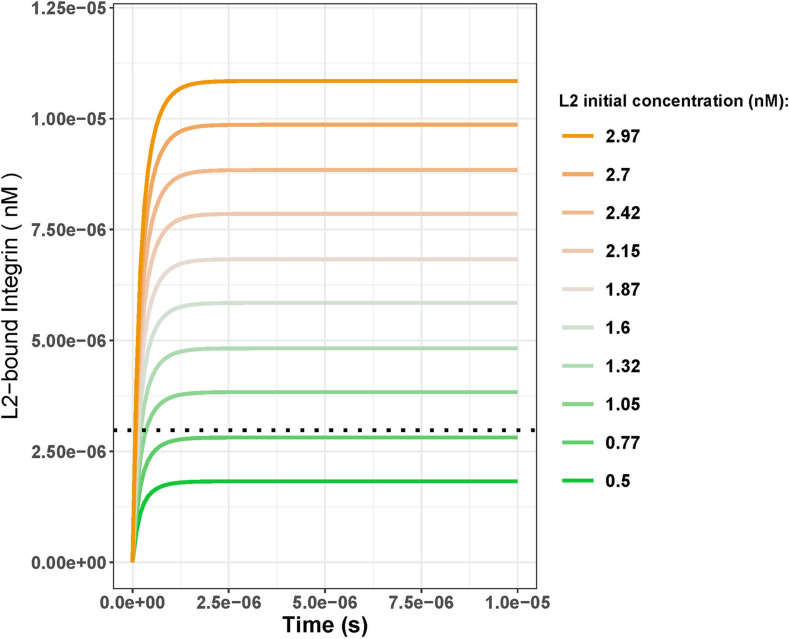
*L*2-bound integrin concentration over time with varying initial concentrations of *L*2 with the initial concentration of *L*1 kept constant at 0.84 nM. Black dotted line shows the day 18 steady-state concentration for *L*2-bound integrins when the initial *L*1 and *L*2 concentrations were both 0.33 nM. Comparing the steady-state concentrations from the line plots to the black dotted line, initial *L*2 concentrations greater than 0.77 nM result in the steady state of the *L*2-bound integrins exceeding that of day 18 (2.98 nM). This corresponds to a fold change of 2.3 between days 18 and 25.

Next, we simulated a 9-fold change [which was the maximum fold-change observed in kidney organoid ECM mass spectrometry experiments by Geuens et al. (2020a)] between days 18 and 25 (Figure 2 condition 4) for *L*2. This simulation was done to reflect the effect of a higher fold of the ligand with the lower integrin binding rate on the system. We saw that with a 9-fold increase in the *L*2 amount on day 25, the steady-state concentration of *L*2-bound integrin was higher than on day 18 (Figure 3B-4) while the *L*1-bound integrin concentration pattern decreased, in the range of 10^–6^ nM, when compared to previous tests (Figure 3A-4 and Supplementary Figure 2D). Mathematically, the decrease in *L*1-bound integrin compensated for the increase in the steady-state concentration of *L*2-bound integrin.

The main observation from simulating test conditions 1 to 4 was that the integrins bound to *L*1 have increased in number by increasing the ligand concentration at day 25 compared to day 18, but integrins bound to *L*2 have not always increased at day 25 with increasing ligand concentration. Only in test condition 4, with a 9-fold increase in the *L*2 amount on day 25, we saw that steady state concentration of *L*2-bound integrins on day 25 was higher than that of day 18.

To further investigate the turning point for the fold change in *L*2, where the *L*2-bound integrin steady-state concentration at day 25 exceeds that of day 18, we ran a parameter scan of the model. For this, we varied the IC for *L*2 (keeping the IC for *L*1 at its day 25 concentration which is 0.84 nM) and compared the *L*2-bound integrin steady state at day 25 to that of day 18 (Figure 4). We ran simulations using 10 different initial concentrations for *L*2 that started from 0.5 nM (1.5-fold increase) and gradually increased to 2.97 nM (9-fold increase). Results showed that, under these parameter settings, the *L*2-bound integrin concentration at day 25 exceeded that at day 18 only when the *L*2 initial concentration was >0.77 nM (2.3–fold greater; Figure 4).

In all tests so far, integrins were bound to *L*2, the ligand with a lower binding rate constant, at a lower concentration than the integrins were bound to *L*1, even when the ICs of the two ligands were equal. This observation implies that the binding rate constants of the two competing ligands, and not the initial concentrations, are of decisive importance for the binding competition.

Thus, we next tested the effect of changing the binding rate constants for *L*1 and *L*2 (Figure 2 and Table 2, condition 5). When we set the binding and unbinding rate constants of the two ligands equal [*k*3 = *k*5 = 1.6 × 10^8^ 1/(nM × s) for binding and (*k*4 = *k*6 = 3.5 × 10^–1^ 1/s for unbinding] and used the same initial concentrations as in condition 1 (Table 2, *L*1 = 0.18 nM, *L*2 = 0.33 nM at day 18 and *L*1 = 0.46 nM, *L*2 = 0.50 nM at day 25), we observed *L*1- and *L*2-bound integrin concentrations to be similar at the steady state (Figures 3C,D). At day 18, the steady-state concentrations for *L*1 and *L*2 were 0.009 and 0.015 nM, respectively. With day 25 conditions, the steady-state concentrations of *L*1- and *L*2-bound integrins were 0.011 and 0.012 nM, respectively.

### The Ligand Binding of Integrin Clusters Reflects the Results of the Ligand Competition

**FIGURE 5:**
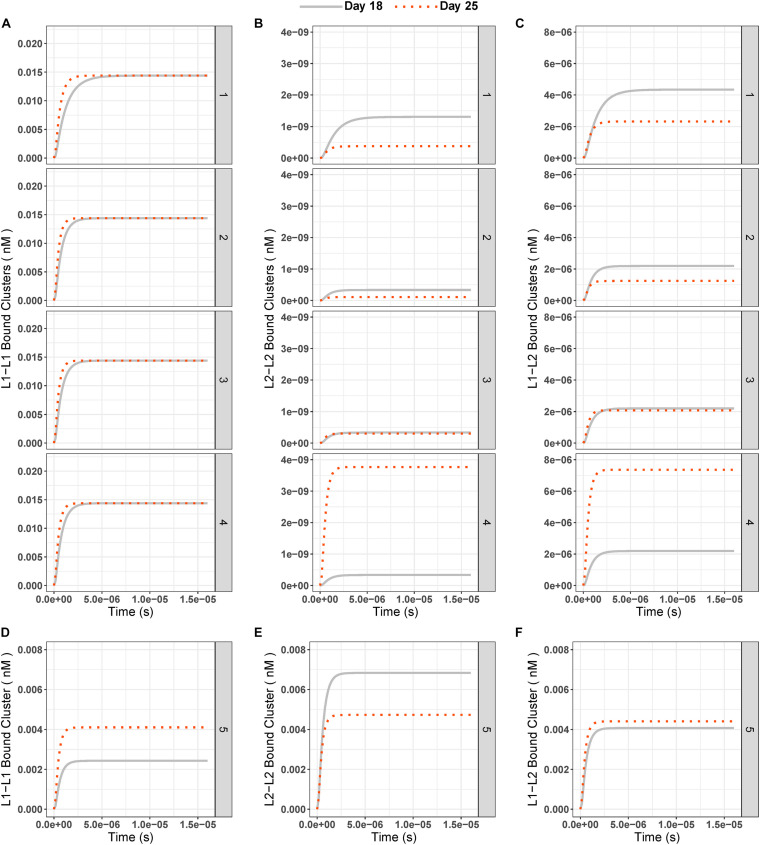
**(A–C)** Concentrations of ligand-bound integrin clusters *L*1–*L*1 (left column, **A-1** to **A-4)**, *L*2–*L*2 (middle column, **B-1** to **B-4)**, and *L*1–*L*2 (right column, **C-1** to **C-4)** over time at days 18 (gray solid line) and 25 (red dotted line) for all test conditions (1–5). **(D–F)** Integrin cluster concentration over time for condition 5 (Different IC (*L*1 = 0.18 nM, *L*2 = 0.33 nM) and equal binding rate constants (*k*3 = *k*5 = 1.6 × 10^8^ 1/nM × s, *k*4 = *k*6 = 3.5 × 10^– 1^ 1/s) for ligands).

Next, we looked at the changes in integrin cluster (*L*1–*L*1, *L*1–*L*2, and *L*2–*L*2) concentrations over time. With different and equal initial conditions on day 18 and respective increases on day 25 (*L*1: 2.5-fold, *L*2: 1. 5-, 2. 5-, and 9-fold), the composition of integrin clusters always reflected the effect of the ligand competition on integrin–ligand binding (Figure 5). In other words, there were always more *L*1-bound, integrin-containing clusters (Figure 5A) at the steady state than *L*2-bound, integrin-containing clusters (Figure 5B), or mixed *L*1–*L*2-bound integrin clusters (Figure 5C). This showed that *L*1 with a higher binding rate is dominant over *L*2 in the clustering step.

When the two cluster species that contain *L*2-bound integrins were compared, we saw that the mixed *L*1–*L*2–bound integrin clusters were higher in concentration at the steady state than *L*2–*L*2–bound integrin clusters (Figures 5B,C) at all times. When day 25 results were compared to day 18, we saw the same pattern as for individual ligand-bound integrin species, namely:

1) *L*1-bound clusters had slightly higher steady state at day 25 than day 18;
2) The *L*2-bound, integrin-containing clusters had lower steady state concentrations at day 25 than day 18 unless the fold change of the ligand between two experiments exceeded 2.3-fold.

Similar to the results of ligand-bound integrins when the same binding-unbinding rates were used for both ligands, clusters with only *L*2-bound integrins were highest in concentration (0.007 nM), followed by *L*1–*L*2–bound mixed integrin clusters (0.004 nM) and *L*1–*L*1–bound integrin clusters (0.002 nM; Figures 5D–F) on day 18. On day 25, with the same settings, the concentrations of the three different integrin clusters were similar: *L*1–*L*1 cluster = 0.004, *L*2–*L*2 cluster = 0.005, and *L*1–*L*2 cluster = 0.004 nM (Figures 5D–F).

### Local Sensitivity Analysis

Finally, we studied the sensitivity of each molecular species in the ligand competition model to changes in model parameters. We performed a local sensitivity analysis by increasing or decreasing by 20%, one of the model parameters at a time. We tested the individual effect of each of the eight binding rate constants (*k*1–*k*8), the initial concentrations of integrins (*i*) and the two competing ligands (*L*1, *L*2) on the steady state of each molecular species in the model. We used these steady-state values for each molecular species to calculate a parameter sensitivity value using the following formula:


$$\mathrm{Parameter}\,\mathrm{Sensitivity}=\frac{\left|S\,S\,\left(k+\Delta \,k\right)-S\,S\,\left(k\right)\right|}{S\,S\,\left(k\right)}\,/\,\frac{\Delta \,k}{k}$$

**FIGURE 6:**
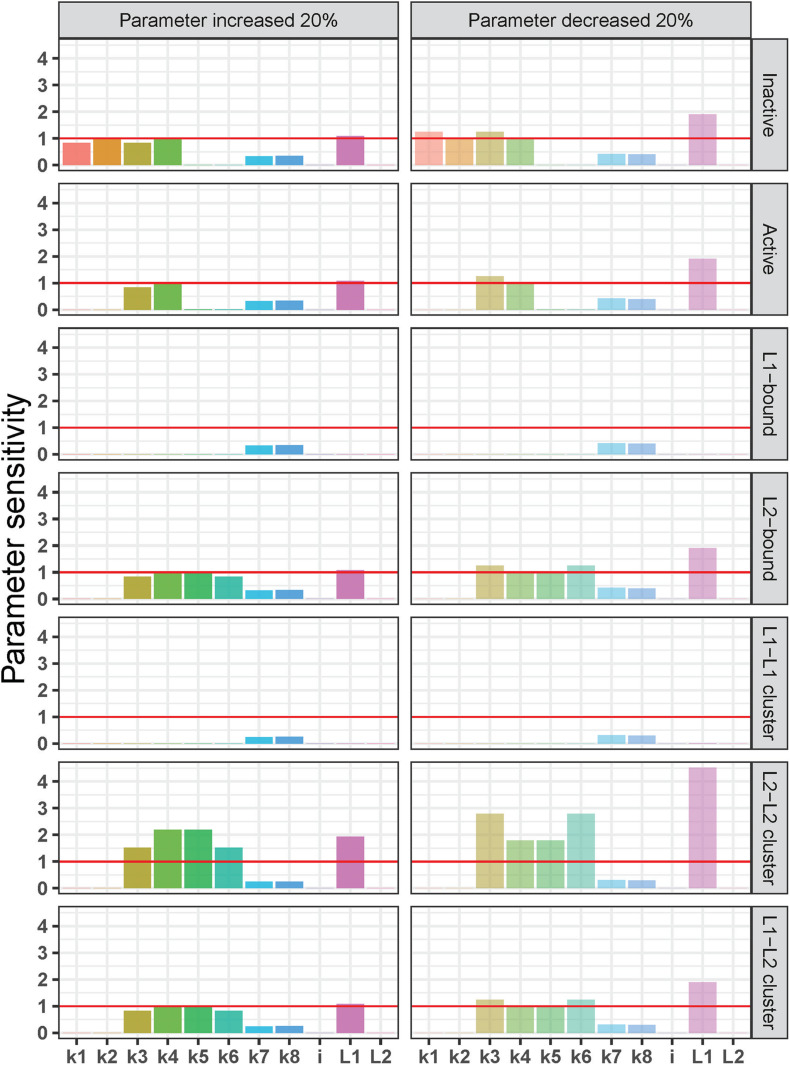
Local sensitivity analysis of the integrin ligand competition model. Parameter sensitivity values on the *y*-axis indicate how a 20% increase (left) or decrease (right) in each parameter [rate constants for integrin activation-inactivation (*k*1–*k*2), *L*1 binding-unbinding (*k*3–*k*4), *L*2 binding unbinding (*k*5–*k*6), integrin cluster formation-dissociation (*k*7–*k*8), and initial concentrations of integrins (*i*) and the two ligands (*L*1 and *L*2)] affects the steady state of each molecular species in the model (from top to bottom: inactive and active integrins, *L*1-bound integrins, *L*2-bound integrins, *L*1-*L* 1-, *L*2–*L* 2-, and *L*1–*L*2-bound integrin clusters). Parameter sensitivity of >1 (red horizontal lines) indicates that the steady state of a molecular species is highly dependent on changes in parameter values, and values < 1 indicates a lower sensitivity to changes in parameter values.

*SS*(*k*) represents the steady-state concentration when there is no change to the model parameter (i.e., the standard model outcome), *SS*(*k* + Δ*k*) represents the steady-state concentration when the parameter value was increased by 20% of the base value. Therefore, $\frac{\Delta \,k}{k}$ was 20% for our analysis. The effect of decreasing the parameter values by 20% was calculated in the same way, replacing *SS*(*k* + Δ*k*) with *SS*(*k*−Δ*k*). The results of this sensitivity analysis are given in Figure 6.

In general, the sensitivity pattern for a decrease in parameter values was the same as for an increase. Similar to the previous tests, *L*1-bound integrins, and *L*1–*L*1–bound integrin clusters were the least affected by changes in model parameters. In contrast to the *L*1-bound integrin concentration, the steady-state, *L*2-bound integrin concentration was not only influenced by the *L*2 binding and unbinding rate constants (*k*5 and *k*6), but also by the *L*1 binding and unbinding rate constants (*k*3 and *k*4) as well as the initial *L*1 concentration. It is noteworthy that the *L*2-bound integrin concentration was affected by the changes in the initial amount of *L*1 and not by the *L*2 increase or decrease.

The *L*2–*L*2–bound integrin cluster was the most sensitive molecular species in the model (Figure 6). We observed that parameter changes that affected *L*2-bound integrins affected also the *L*2–*L*2–bound integrin clusters but in a more dramatic way: the steady state of *L*2–*L*2–bound integrin cluster was more than twice as sensitive to the 20% decrease in *L*1 initial concentration than that of the *L*2-bound integrin. A similar pattern was observed for the 20% increase in *k*4 (*L*1 unbinding) and *k*5 (*L*2 binding) as well as for the 20% decrease in *k*3 (*L*1 binding) and *k*6 (*L*2 unbinding). Interestingly, the mixed *L*1–*L*2–bound integrin clusters showed a very similar sensitivity pattern to *L*2-bound integrins, which is different from that of *L*2–*L*2-bound integrin clusters. These observations can be explained by the quadratic dependency of *L*2–*L*2-bound integrin clusters to *L*2-bound integrins (detailed in the section “Discussion”).

We should also note that both the steady state of inactive and active integrins did not show significant sensitivity to the changes in the initial concentration of inactive integrins (*i*) but were sensitive to the changes in *L*1 initial concentration (*L*1; Figure 6). This is expected given that the amount of ligands in the system is in excess compared to integrin concentration (*i* = 0.05 nM, *L*1 = 0.18 nm, and *L*2 = 0.33 nM) to mimic the biology of receptor–ligand binding (Wanant and Quon, 2000; Hudson et al., 2017). The excess ligand concentrations ensure that with the original reaction rate constants and in all the test scenarios, the unbound integrins in the system (both active and inactive) reach a steady-state concentration close to zero and are found as ligand-bound (Supplementary Figure 1). The parameter sensitivity patterns of inactive and active integrins show that the imbalance between integrin–ligand amounts is maintained by *L*1, the ligand with higher binding affinity. It is the *L*1 initial concentration and its ligand binding-unbinding constants (*k*3, *k*4) that affect the steady-state concentrations of inactive and active integrins in the parameter sensitivity analysis, while the *L*2 initial concentration and its binding rates (*k*5, *k*6) are not determinants (Figure 6).

In line with this, when we set the initial integrin concentration to be higher (1 nM) than the total initial ligand concentration (0.51 nM), we observed a major shift in the parameter sensitivity patterns (Supplementary Figure 3). When the integrins were in excess compared to ligands, the steady states of the integrin molecules in the system were highly dependent on the initial integrin concentration (Supplementary Figure 3) instead of on the binding-unbinding constants when the ligands were in excess. This is expected as all ligands will be bound to the receptors in a system where there are more receptors than ligands. The amounts of ligand-bound receptors therefore correlate with the initial ligand concentrations.

## Discussion

Here we present an ODE model that can be used to explore the ligand-binding kinetics of integrins. The computational model involves three main biological reactions: (1) integrin activation, (2) integrin–ligand binding, and (3) ligand-bound integrin clustering (Figure 1). At each step, the model allows us to track the concentration of each model species over time. Different from previously published models of integrin–ligand binding (Macdonald et al., 2008; Hudson et al., 2017; Yu et al., 2017), we included two ligands that have the ability to bind to the same type of integrin, which allowed us to monitor the competition between these two ligands.

The first outcome of the different tests we performed using the ODE model was that all integrins were activated, bound to a ligand, and that a subset of these ligand-bound integrins were clustered (Supplementary Figure 1). Biologically, integrin activation happens in two ways: outside-in and inside-out. Outside–in activation is triggered by the interactions between integrins and ECM ligands while inside-out activation is triggered by the binding of talin to cytoplasmic tails of integrin molecule (Shams et al., 2017). Talin is a protein that harbors multiple binding sites for other signaling molecules and the actin cytoskeleton (Miller et al., 2020). Both these activation processes are not very well resolved but it is known that they influence one another (Shams et al., 2017). The activation step in our model is not specific to either of the above activation mechanisms.

Our parameter sensitivity results indicated that the rates of integrin activation/inactivation did not have a significant impact on the steady-state concentrations of molecular species involved in reactions like ligand binding or integrin clustering (Figure 6). We can speculate that in a model that includes multiple interaction partners of integrins which affect the integrin activation or reactions of downstream signaling pathways which are dependent on the integrin activation, the integrin activation step would play a significant role in the model. It is known that for example, talin-mediated integrin activation is dynamically regulated by several potential mechanisms (Calderwood, 2004) such as talin proteolysis (Yan et al., 2001) and competition between other proteins that bind to integrin from its cytosolic tails (Bouvard et al., 2003). Yet the exact mechanisms of action and their relative significance are not resolved (Calderwood, 2004). Exploring these mechanisms and their effects on ligand binding and integrin clustering could be one potential extension to our model.

We used the results from a proteomics analysis of kidney organoid ECM when defining the ligand concentrations in our model simulations. Under these “experimental conditions,” we observed that the steady-state concentration of *L*1-bound integrins was higher than the *L*2-bound integrins concentration (0.021 nM vs 6.393 × 10^–6^ nM, Figures 3A-1,B-1), even though the initial concentration of the *L*2 ligand is greater than *L*1 on day 25 (0.50 vs 0.46 nM, Table 2). We observed this pattern even when we systematically changed the initial concentrations of the two ligands and the concentration fold changes between days 18 and 25.

In their ODE model, Hudson et al. (2017) also reported an increase in the ligand-bound integrin amount whenever there was in increase in the ligand concentration. However, their model included only one type of ligand binding to integrin at a time. When we account for ligand competition, we observed that an increase in ligand concentration did not ensure more ligand-bound integrins if the competing ligand has a higher binding rate (Figure 3). For the standard parameter settings, only fold changes greater than 2.3 led to an increase in *L*2-bound integrin amounts (for 0.84 nM *L*1 at day 25, Figure 4). However, even with high fold changes (9-fold compared to 2.5-fold), *L*1-bound integrins were always more abundant than *L*2-bound integrins at the steady state.

We can explain this observation, where an increase in (initial) ligand concentration results in a reduction of its integrin-bound, steady-state value, using the ODE system given in Eqs 1–9. When at steady state, all ODE equations should be equal to zero because there is no time-dependent change in the concentrations of any of the molecular species. Using the steady state solutions of Eqs 8, 9, we can get to the following dependencies between ligand-bound integrin concentrations at the steady state (*IL*1*s* and *IL*2*s*) and ligand concentrations at the steady state (*L*1*s* and *L*2*s*);


(10) k⁢3×I⁢s×L⁢1⁢s=k⁢4×I⁢L⁢1⁢s

(11) $$I\,L\,1\,s=\frac{k\,3\times I\,s\times L\,1\,s}{k\,4}$$

(12) k⁢5×I⁢s×L⁢2⁢s=k⁢6×I⁢L⁢2⁢s

(13) $$I\,L\,2\,s=\frac{k\,5\times I\,s\times L\,2\,s}{k\,6}$$

(14) $$\frac{I\,L\,1\,s}{I\,L\,2\,s}=\frac{k\,3\times k\,6\times L\,1\,s}{k\,4\times k\,5\times L\,2\,s}$$

In our system, *k*3 = 1.6 × 10^8^ 1/(nM × s) and *k*4 = 3.5 × 10^–1^ 1/s while *k*5 = 1.6 × 10^4^ 1/(nM × s) and *k*6 = 2.3 × 10^–2^ 1/s. When plugged in to Eq. 14, these rate constants provide *IL1s* to be 660 times *IL2s*. The difference between the steady-state concentrations of the ligands, however, is not high enough to compensate for the big difference in rate constants, resulting in a big difference between steady-state concentrations of the two types of ligand-bound integrins. This also explains the differences in the sensitivity patterns of ligand-bound integrin species. The steady state of integrins bound to *L*1 with a higher binding rate constant (*IL*1) is less affected by the small perturbations in model parameters compared to *L*2-bound integrins (*IL*2; Figure 6), because $\frac{k\,3}{k\,4}$ is big enough to compensate for a 20% change. From these results, we can conclude that in case of ligand competition for a receptor, the highest ratio — either the ratio of binding rate constants or the ratio of initial ligand concentrations — has the dominating effect on the steady-state concentrations of the ligand-bound receptors.

When the binding and unbinding rate constants of the two ligands are set to be equal (i.e., *k3* = *k4* and *k5* = *k6*), we can see from Eqs 11, 13 that the difference between steady-state concentrations of the ligand-bound integrins (*IL1s* and *IL2s*) solely depend on the difference between the steady-state concentrations of the two competing ligands (*L1s* and *L2s*). Since we assume mass conservation in the system, the following equations hold true for the total amount of ligands in the system at the steady state:


(15) L⁢1=L⁢1⁢s+2×C⁢1⁢s+C⁢3⁢s+I⁢L⁢1⁢s

(16) L⁢2=L⁢2⁢s+2×C⁢2⁢s+C⁢3⁢s+I⁢L⁢2⁢s

*L1* and *L2* represent the initial (and total) ligand concentrations in the system. As all the rate constants for forward and reverse reactions become equal for the scenario where the two ligands have equal binding rate constants, we can safely assume that the ligand concentrations at the steady state correlate with the initial ligand concentrations.

Using Eqs 14–16, we can explain what we observe in Figures 3C,D, i.e., for day 18 initial conditions of the two ligands with equal binding rates, the ratio of the steady-state concentration of *L*1-bound integrins (*IL*1) to that of *L*2-bound integrins (*IL*2) is 0.59 (0.009 nM/0.015 nM). This ratio is very similar to the ratio of initial *L*1 amount to initial *L*2 amount, which is 0.54 (0.18 nM/0.33 nM). When we look at the day 25 steady-state concentrations, we find the *IL*1/*IL*2 ratio to be 0.93 (0.011 nM/0.012 nM) and an initial ligand concentration ratio of 0.92. Therefore, the initial ligand concentrations, when the binding rate constants are equal, are informative for predicting the steady-state concentrations of ligand-bound integrins. In other words, with equal binding rates, the ligand with the highest initial condition will result in the highest integrin-bound, steady-state concentration and “win” the ligand competition. This observation is also in line with the literature. In another partial differential equation model of competitive receptor–ligand binding, the competing ligands both had binding affinities in the picomolar range and the steady-state concentrations of receptors bound to either of the ligand were directly correlated with the initial ligand concentrations (Mac Gabhann and Popel, 2004).

These explanations of the relationship between the binding affinities of competing ligands and the final amount of ligand-bound integrins can be the mathematical explanation of the experimental finding in which RGD peptides with different stereochemistry inhibit the binding of a subset of integrin ligands, while being ineffective for inhibiting other ligands. For example, one of the very early studies on cyclic vs linear RGD peptides reported that the peptides could inhibit vitronectin binding effectively while falling short on inhibiting fibronectin binding (Pierschbacher and Ruoslahti, 1987). This was because the peptide constructs had a larger affinity for the integrins compared to the affinity of vitronectin for the integrins while fibronectin still had the highest affinity for the integrins therefore the peptide constructs failed to inhibit the adhesion to fibronectin. Ever since, many others developed integrin targeting peptides with various binding affinity and selectivity (Wang et al., 2005; Kimura et al., 2009; Mas-Moruno et al., 2011; Piras et al., 2012; Bernhagen et al., 2017; Ma et al., 2017). Although our computational results do not point out to a solution on how to improve the affinity of a ligand toward an integrin, we provide here a method for calculating the effect of having a higher affinity ligand on the binding of other competing ligands. This can be used to estimate the affinity that needs to be reached to prevent the binding of a specific competitive ligand, without having to run a series of experiments with a large set of ligands, different concentrations, and/or timing.

As expected, due the same clustering rates *k*7–*k*8 for all cluster-types, the steady-state composition of ligand-bound integrin clusters (*IL*1–*IL*1, *IL*2–*IL*2, and *IL*1–*IL*2, Figure 1) was in correlation with the steady-state concentrations of single, ligand-bound integrins (Figures 3, 5). *L*1-bound, integrin-containing clusters were in abundance when compared to *L*2-bound, integrin-containing clusters, except when the binding rate constants were set to be equal for both ligands (Figures 5C,D). We can also explain this observation analytically by setting Eqs 11–13 to zero to calculate the steady state of the three integrin cluster species. Then we obtain the following equations:


(17) $$C\,1\,s=\frac{k\,7\times I\,L\,1\,s^{2}}{k\,8}$$

(18) $$C\,2\,s=\frac{k\,7\times I\,L\,2\,s^{2}}{k\,8}$$

(19) $$C\,3\,s=\frac{k\,7\times I\,L\,1\,s\times I\,L\,2\,s}{k\,8}$$

where *C*1*s*,*C*2*s*, and *C3s* denote the steady-state concentrations of the three integrin cluster species composed of *IL*1–*IL*1, *IL*2–*IL*2, and *IL*1–*IL*2, respectively. Eqs 17–19 reveal that the steady-state concentrations of all integrin clusters correlate with the steady-state concentrations of the ligand-bound integrin concentrations that they contain. Because of the quadratic term in Eq 18, the steady state of *IL*2–*IL*2 clusters (*C*2) is much more sensitive to small perturbations in model parameters than the steady state of *L*2-bound integrins (Figure 6). In contrast, the relatively high steady state value of *L*1-bound integrins balances the steady state of *IL*1-containing clusters (*C*1 and *C*3), therefore their sensitivity patterns are similar that of *IL*1 and *IL*2, respectively (Figure 6).

We should note that the integrin clusters in our model were composed only of two ligand-bound integrins, whereas in reality this number can be much higher. Previous models of integrin clustering suggest that as the ligand-binding rates increase, the size of integrin clusters decrease, possibly due to the decreased diffusion rate of ligand-bound integrins (Cheng et al., 2020). Neither the exact number of possible integrins in a cluster nor the effect of the composition of integrin clusters is known. Since it is known that at focal adhesion points, more than one type of integrin can cluster together and they have different roles in the cluster (Roca-Cusachs et al., 2009), it would be interesting to explore the downstream effects of having different ligand-bound integrins clustered together.

For the sake of simplicity and interpretability of the ligand competition, we assumed the spatial distribution of molecules to be homogenous in this model. Therefore, we used an ODE model and assumed the free ligands are always available to active integrins, independent of their spatial location. However, previous models with a focus on integrin clustering have suggested a limit to the distance between ECM ligands for the integrin clustering to occur (Jamali et al., 2013; Yu et al., 2017). Therefore, future models should focus on including the space dimension to integrin–ligand binding and clustering models, considering ligand spacing.

In natural tissues, there can be more than two ligands competing to bind to the same integrin. Therefore, our model is a simplified version of the real scenario. Nevertheless, we have shown that even with this simplified ligand-competition model, we can acquire more fundamental understanding of integrin–ligand binding. For example, we have shown that the vWA-bound integrins (*IL*2) are much lower in concentration than fibronectin-bound integrins (*IL*1) when the two ligands are allowed to bind simultaneously, in contrast to the model results of Hudson et al. (2017). In addition, our model suggests that with an increasing number of ligands competing for the same integrin, the final distribution of ligand-bound integrins will correlate with the distribution of their binding affinities. When we added a third ligand to our system (*L*3), with a binding affinity even higher than *L*1, we observed that the number of *L*3-bound integrins were higher than both *L*1- and *L*2-bound integrins (Supplementary Figure 4). Whereas *L*1-bound integrins were still more abundant than *L*2-bound integrins (Supplementary Figure 4).

It would be interesting to expand the current model with different integrin types, to reflect the level of complexity of interactions at the cell–ECM interface. However, this would increase the number of parameters in the model and to date, binding rates for all integrin–ligand pairs are not known. Such future work should focus on obtaining binding rate constants specific to different integrin–ligand pairs. Surface plasmon resonance (Yan et al., 2001; Kim et al., 2005; Elosegui-Artola et al., 2014) or single molecule dynamic force spectroscopy (Taubenberger et al., 2007) are suitable techniques for this purpose. The current model focuses on the short-term behavior, neglecting ligand production and downstream signaling. As such, another interesting avenue to expand the ligand-competition model could be to include downstream cytosolic events from the ligand-bound integrins that alters the cell behavior. In the end, this would lead to the prediction of cell behavior using the information on the ECM composition.

In summary, with our model, we conclude that the control over the concentrations of ECM ligands would not be enough to have control over their integrin binding in case there is a significant difference between the binding rates of different ECM ligands. More specifically, our results show that, for the low-affinity ligand, not only its ligand binding-unbinding rates are important, but also the ligand binding-unbinding rates and initial concentration of the competing ligand with a faster binding rate (Figure 6). In light of this information, the increased production of ligands with higher binding affinity would disable lower-affinity ligands from binding to integrins. In cases where biochemical cues from slower binding ligands are needed for the healthy development of cells in culture, their development would be disrupted. This could be the root cause, for example, of persistent challenges in functional kidney organoid development field such as off-target cell populations, lack of vascularization and insufficient maturation introduced in prior sections (Nishinakamura, 2019; Geuens et al., 2020b).

To overcome such effect, either the binding of faster binding ligands needs to be impaired by blocking agents, or the cellular production of faster binding ligands needs to be prevented using molecular biology techniques. Alternatively, synthetic integrin ligands with controlled affinity could be used to selectively prevent binding of naturally produced ECM proteins. Of course, these preventive strategies require a thorough understanding of the integrin function, cellular signaling and decision-making affected by ligand-integrin interactions. Experimental biology going hand-in-hand with computational biology can answer many unknowns in the understanding of integrins (Karagöz et al., 2021).

As such, this study shows that computational models can be informative to get a better understanding of the effects of ECM composition on the cell behavior and to develop cell culture conditions that would favor desired cell phenotypes. Moreover, since our model fundamentally explains a reaction system, in which there are two ligands available to bind to their receptor, the obtained relations and influential factors describing ligand competition are generic and applicable to other receptor–ligand interactions.

## Data Availability Statement

The datasets presented in this study can be found in online repositories. The names of the repository/repositories and accession number(s) can be found below: https://github.com/zeynepkaragoz/Ligand_competition_model.

## Author Contributions

ZK developed the computational model. ZK and AC analyzed its results. ZK, TG, MG, and AC contributed to the design and conception of the study. ZK drafted the manuscript. All authors contributed to manuscript revision, read, and approved the submitted version.

## Conflict of Interest

The authors declare that the research was conducted in the absence of any commercial or financial relationships that could be construed as a potential conflict of interest.

## Footnotes

1
https://github.com/zeynepkaragoz/Ligand_competition_model
