# Corrigendum: Win, Lose or Tie: Mathematical Modeling of Ligand Competition at the Cell-Extracellular Matrix Interface

**DOI:** 10.3389/fbioe.2021.771429

**Published:** 2021-09-22

**Authors:** Zeynep Karagöz, Thomas Geuens, Vanessa L. S. LaPointe, Martijn van Griensven, Aurélie Carlier

**Affiliations:** Department of Cell Biology–Inspired Tissue Engineering, MERLN Institute for Technology-Inspired Regenerative Medicine, Maastricht University, Maastricht, Netherlands

**Keywords:** integrin, ligand competition, computational model, extracellular matrix, ordinary differential equation

In the published article, the “Parameter Sensitivity” formula was erroneously given as:

$$Parameter\;Sensitivity\;=\;\frac{\left|SS\left(k+\Delta k\right)-SS\left(k\right)\right|}{SS\left(k+\Delta k\right)}\;/\frac{\Delta k}{k}$$

However, the correct “Parameter Sensitivity” formula in the first paragraph of the **Results** section entitled “*Local Sensitivity Analysis*” should instead read as:

$$Parameter\;Sensitivity\;=\;\frac{\left|SS\left(k+\Delta k\right)-SS\left(k\right)\right|}{SS\left(k\right)}\;/\frac{\Delta k}{k}$$

The correct formula has been used to perform the calculations reported in the publication.

The authors apologize for this error and state that this does not change the scientific conclusions of the article in any way. The original article has been updated.

