# Supplementary material for: Win, Lose, or Tie: Mathematical Modeling of Ligand Competition at the Cell–Extracellular Matrix Interface
Source: Front Bioeng Biotechnol. 2021 Apr 29;9:657244. doi: 10.3389/fbioe.2021.657244 (PMC8117103; doi:10.3389/fbioe.2021.657244)
Supplement: Supplementary file 1 [file Data_Sheet_1.PDF]

Supplementary Material

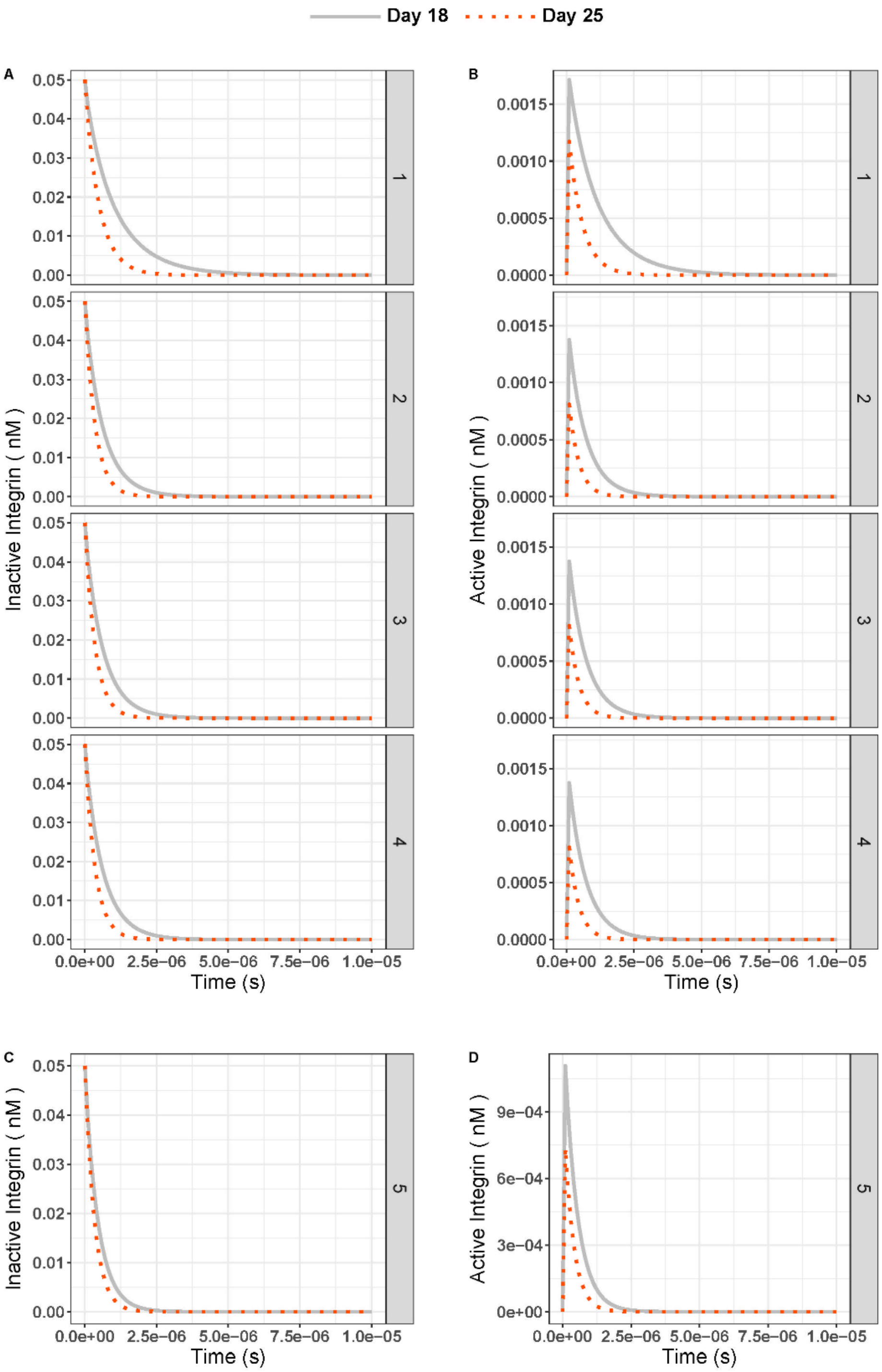

Figure S1: (**A**) Inactive and (**B**) active integrin concentrations over time in experiment days 18 (gray solid line) and 25 (red dotted line) for test conditions 1 to 4. **(C)** Inactive and (**D**) active integrin concentrations over time for test condition 5. The experimental conditions 1 to 5 are as given in Table 2 in the main text.

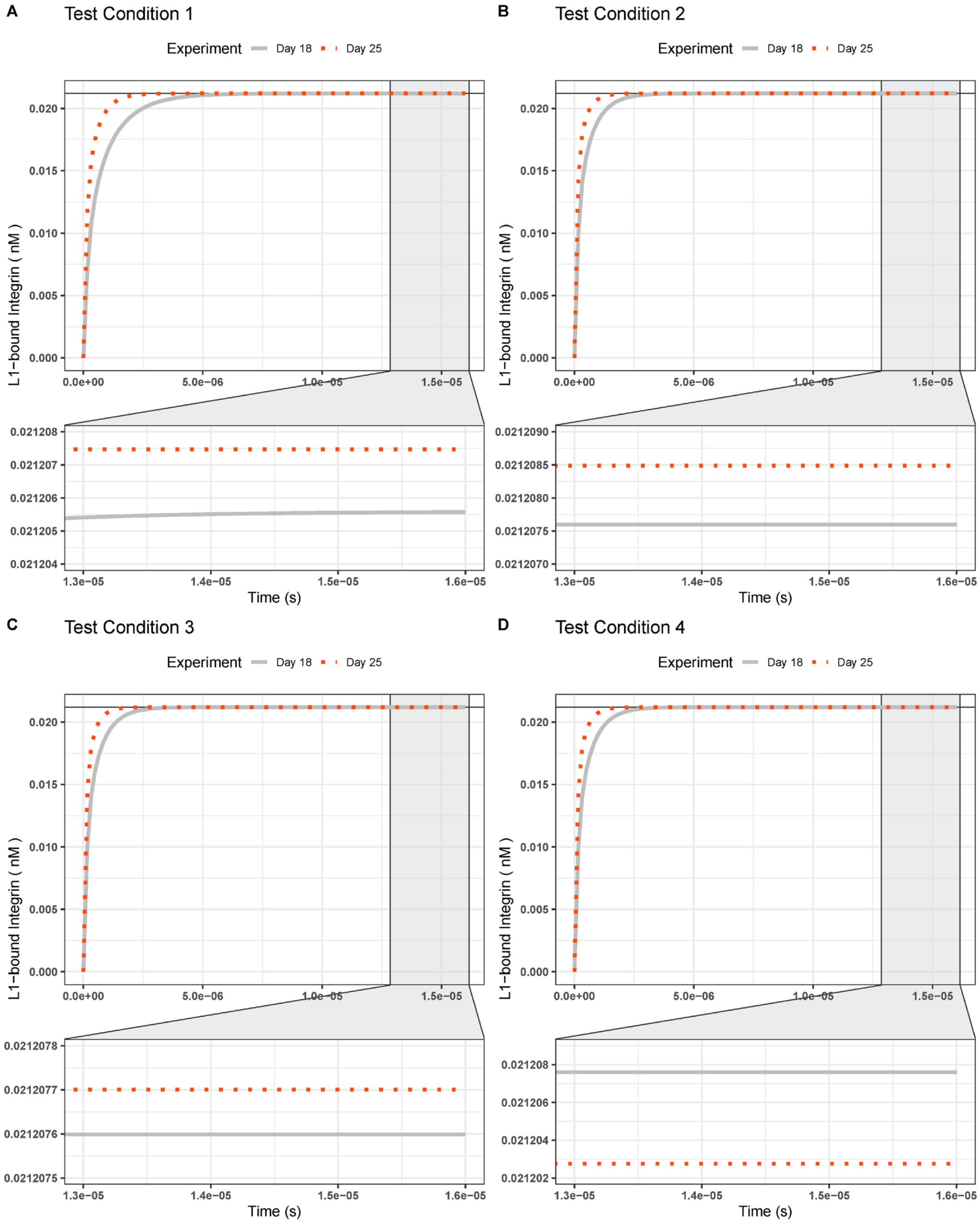

Figure S2: Zoom into the Figure 3 in the main text. An increase was observed in L1-bound integrin steady state concentrations on day 25 compared to day 18 in test cases 1 **(A)**, 2 **(B)** and 3 **(C)**. In test condition 4 however, L2-bound integrin concentration increases on day 25 compared to day 18. This increase is compensated by a decrease in L1-bound integrin concentration **(D)**. Note that in all four test conditions, the L1-bound integrin steady state concentration is higher than the L2-bound integrin steady state concentration.

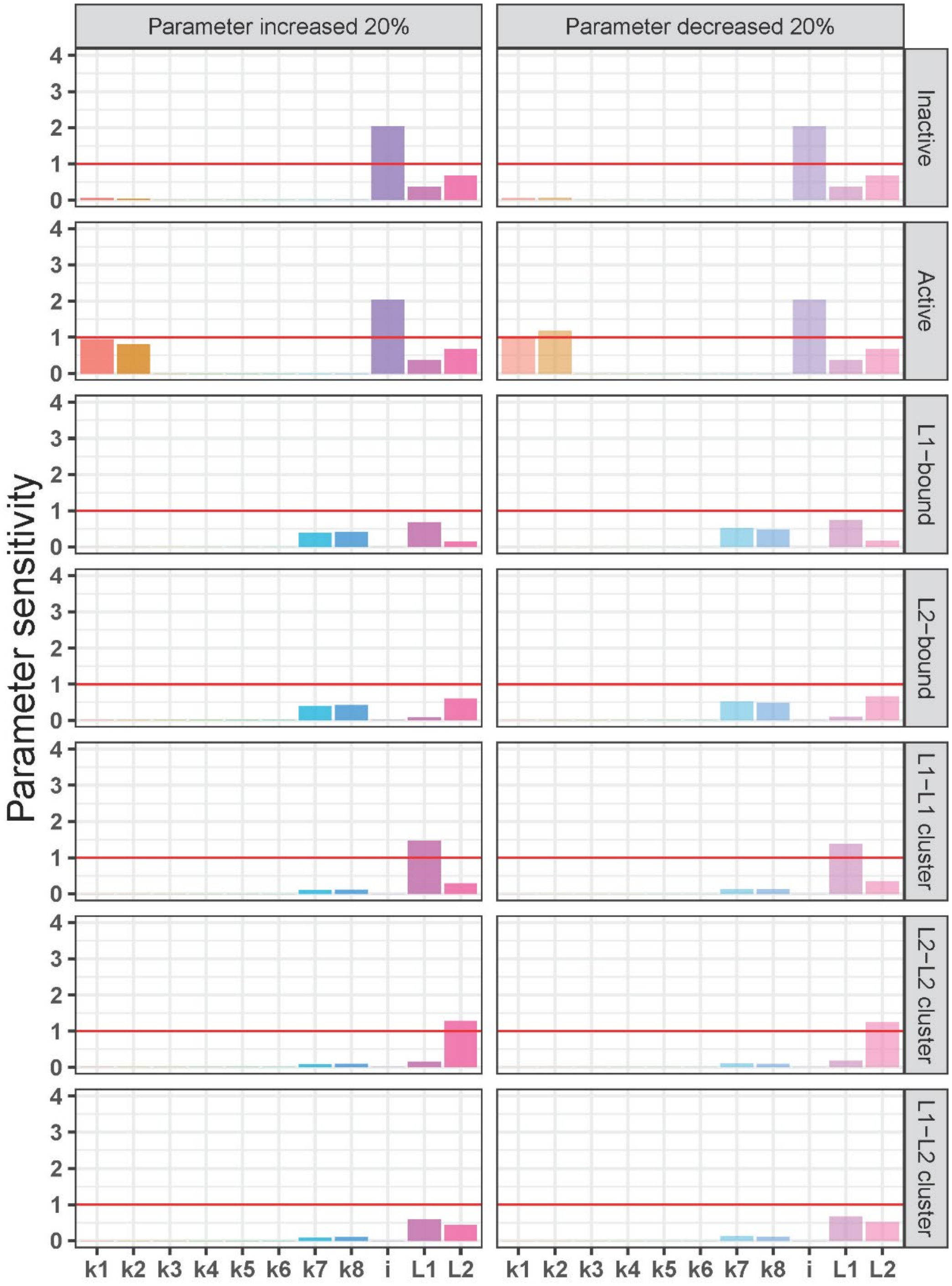

Figure S3: Parameter sensitivity analysis results for the model when the integrin initial concentration was set to be greater (1 nM) than the total initial concentration of the competing ligands (0.51 nM). Compared to the parameter sensitivity of model with original settings, the binding/unbinding rates are not as determinant. The inactive and active integrin steady state is highly dependent on the initial integrin concentration in this model, whereas in the original model, initial integrin concentration was not a determinant factor.

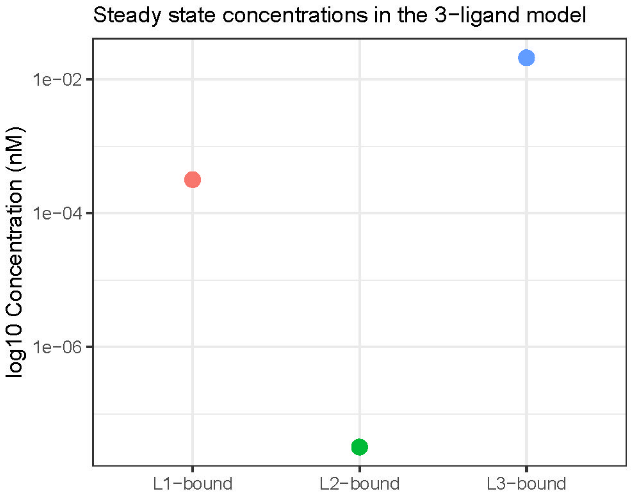

Figure S4: Comparison of steady state concentrations of three ligand-bound integrin species in a theoretical 3-ligand model. We have created a ligand competition model with three ligands, L1 and L2 being the same ligands as in the ligand competition model explained in the main text. L3 was introduced as being the ligand with the highest affinity ($k_{on} = 1.6 \times 10^{10}$ 1/(nM × s), $k_{off} = 2.3 \times 10^{-1}$ 1/s). We ran the simulations for equal initial ligand concentrations for all three ligands (L1 = L2 = L3 = 0.33 nM). The figure shows that the steady state concentrations of the ligand bound integrins depend on the affinity of each ligand towards the integrin. As such, L3-bound integrins were highest in concentration, followed by the L1-bound integrin concentration. The L2-bound integrins were lowest in concentration. Note that L3 in this model is not based on any actual ligand but was only added to the system to demonstrate the case of multiple high affinity ligands present in the binding competition.
